# Molecular Epidemiology of *Clostridioides difficile* Infections in Patients Hospitalized in 2017–2019 at the Central Teaching Hospital of Medical University of Lodz, Central Poland

**DOI:** 10.3390/antibiotics14030219

**Published:** 2025-02-21

**Authors:** Agata Ptaszyńska, Anna Macieja, Dominika Rosińska-Lewandoska, Filip Bielec, Piotr Machnicki, Małgorzata Brauncajs, Dorota Pastuszak-Lewandoska

**Affiliations:** 1 Department of Microbiology and Laboratory Medical Immunology, Medical University of Lodz, 92-213 Lodz, Polandpiotr.machnicki@umed.lodz.pl (P.M.); malgorzata.brauncajs@umed.lodz.pl (M.B.); dorota.pastuszak-lewandoska@umed.lodz.pl (D.P.-L.); 2 Department of Microbiology and Pharmaceutical Biochemistry, Medical University of Lodz, 92-215 Lodz, Poland; anna.macieja@umed.lodz.pl; 3 Medical Microbiology Laboratory, Central Teaching Hospital of Medical University of Lodz, 92-213 Lodz, Poland

**Keywords:** *Clostridioides difficile*, ribotype 027, *vanA* gene, *nim* gene, molecular epidemiology, antimicrobial resistance

## Abstract

**Background/Objectives:** *Clostridioides difficile* infection (CDI) represents a significant public health challenge globally, driven by its increasing prevalence, hypervirulent strains like ribotype 027 (RT027), and growing antibiotic resistance. This study aimed to evaluate the prevalence of RT027 and analyze molecular markers of vancomycin and metronidazole resistance in stool samples from CDI patients hospitalized in Poland between 2017 and 2019. **Methods:** A total of 200 stool samples from confirmed CDI cases were analyzed for the presence of RT027, *vanA* (vancomycin resistance), and *nim* (metronidazole resistance) genes. DNA was extracted, and a polymerase chain reaction (PCR) was conducted using specific primers. Statistical associations between RT027 and resistance genes were evaluated using chi-square tests and logistic regression. **Results:** RT027 was detected in 14% of samples. The *vanA* gene, indicative of vancomycin resistance, was found in 52.5% of samples, while the *nim* gene, associated with metronidazole resistance, was present in 1.5% of cases. Co-occurrence of RT027 with *vanA* was not statistically significant. The study revealed no significant association between RT027 and *vanA*. Also, no significant association was observed between RT027 and *nim* due to the latter’s low prevalence. **Conclusions:** This study highlights a concerning prevalence of *vanA* among CDI cases, indicating widespread vancomycin resistance and challenging current treatment guidelines. While RT027 prevalence was moderate, no significant associations with vancomycin or metronidazole resistance were observed. These findings emphasize the need for molecular surveillance and improved antimicrobial stewardship to manage CDI effectively.

## 1. Introduction

*Clostridioides* spp. is a Gram-positive, relatively anaerobic bacterium that can create terminal spores resistant to environmental factors, allowing it to survive in unfavorable conditions. It is commonly present as a fecal contaminant, and infection occurs primarily through the ingestion of spores, which can persist in the environment for prolonged periods. The bacterium can also produce toxins (A, B, or both) which are the main virulence factors of this pathogen. These toxins trigger an inflammatory response with fluid secretion and tissue damage, ultimately leading to diarrhea. Strains that do not have toxins are nonpathogenic. Additionally, individuals can carry toxigenic and toxin-producing *Clostridioides difficile* without having any infection symptoms, which means they are colonized [1]. Asymptomatic carrier state affects approximately 3% of adults and two-thirds of children [2].

*C. difficile* infection (CDI) has been defined by the Infectious Diseases Society of America (IDSA) and the Society for Healthcare Epidemiology of America (SHEA) as three or more unformed stools in 24 h with a positive stool toxin and a positive result of the nucleic acid amplification test or the glutamate dehydrogenase test [3]. The clinical picture of CDI ranges from mild self-limiting diarrhea to symptoms of toxic megacolon (*megacolon toxicum*) [2]. Fast and correct diagnosis is essential for optimal patient care and preventing the bacteria from spreading.

*C. difficile* is the principal etiologic factor responsible for diarrhea related to antibiotic therapy in hospitals and is recognized as a major healthcare-acquired infection (HAI) pathogen. Its spores can persist in the hospital environment and be transferred to patients from the hands of healthcare personnel who have touched a contaminated surface or item [4]. Nowadays, it contributes to significant medical and economic burdens in hospital departments. In Poland, the CDI incidence in 2022 was 55.4 out of 100,000 people (21,371 cases), which was similar to 2021 but double the number from 2020, according to the National Institute of Hygiene Reports, the Department of Infectious Disease Epidemiology and Surveillance, and the Laboratory of Monitoring and Analysis of Epidemiological Situations [5]. The European Center for Disease Prevention and Control (ECDC) points out that *C. difficile* ranks eighth among microorganisms that cause hospital infections [6]. The research conducted between 2011 and 2013 showed that CDIs in 13 Polish hospitals were induced mainly by *C. difficile* ribotype 027 (RT027), 62.9% in 2012 and 61.8% in 2013 [7]. The newest data from 2017 has shown the occurrence of RT027 in about 82.4% hospitals in Silesia, Poland [8].

Ribotypes are molecular subtypes of *C. difficile* distinguished based on the patterns of ribosomal RNA (rRNA) genes. These ribotypes allow researchers and clinicians to classify bacterial strains, which can differ in virulence, antibiotic resistance, and prevalence [9]. *C. difficile* ribotypes can be categorized into two main groups: epidemic (hypervirulent) and non-epidemic strains (see Table 1).

The hypervirulent strain RT027 has gained importance since the first outbreaks associated with it: first in Canada and the USA (2003–2004), and later in Europe (e.g., 2004–2006 in the UK, 2009–2012 in Italy). In 2010–2015, this strain was dominant in many hospitals worldwide [13]. It has been revealed that patients above 65 years old are three times more likely to have strain RT027 than patients below this age [14]. Moreover, RT027 causes the most severe disease, with a higher risk of acute complications, recurrences, and even death. The infection with this type of bacteria is associated with two to three times higher risk of death in the elderly [15]. Mortality fluctuates from 2% to 6%. It is higher in patients with inflammatory bowel disease or admitted to intensive care units [16,17].

According to the 2012 research, the incidence of RT027 in the UK has decreased from 55% to 21%. The reason could be a reduction of fluoroquinolones and better healthcare personnel awareness [18]. Notably, during 2016–2017, RT027 was highly prevalent in Hungary (67.6% of cases), Poland (63.0%), and Slovenia (44.4%), while other countries reported a prevalence of approximately 2.5% [19]. A 2024 study highlighted that RT027 and the emerging RT181 exhibited elevated minimum inhibitory concentrations to moxifloxacin, clindamycin, and rifampicin, indicating increased antimicrobial resistance. This resistance was particularly pronounced in Eastern Europe, whereas isolates from Northern and Western Europe showed lower resistance levels [20].

Toxin A and toxin B, produced by *C. difficile*, are encoded by *tcdA* and *tcdB* genes located within a locus of pathogenicity (PaLoc). The other genes located at this locus, i.e., *tcdR*, *tcdE*, *tcdL*, *tcdC*, are implicated in toxin expression and secretion. RT027 is characterized by an 18 bp deletion and a single nucleotide deletion at position 117 in the *tcdC* gene, resulting in the inactivation of the toxin repressor *tcdC* and higher amounts of toxin production [11,21].

**Figure 1:**
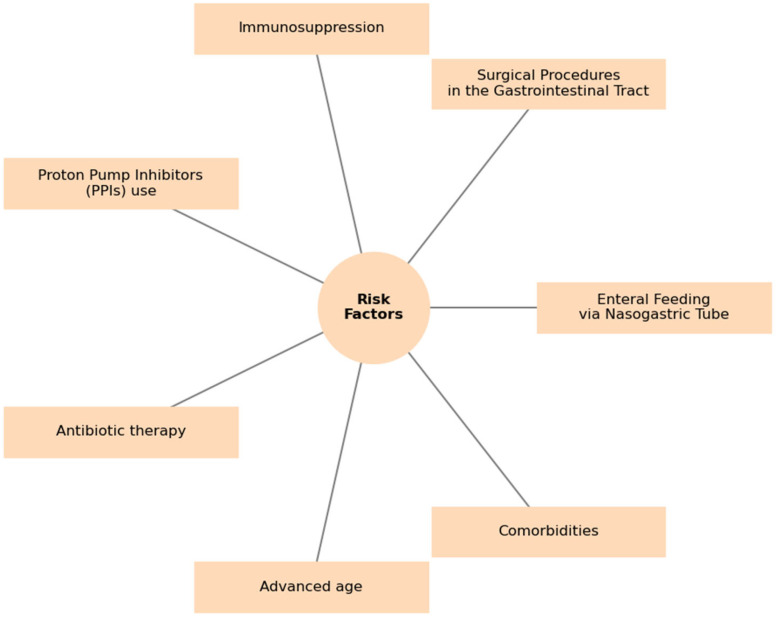
Main risk factors for CDI [13,25].

Several factors lead to the CDI. The first and the most significant is antibiotic therapy, that disturbs gut microbiota. Clindamycin, fluoroquinolones, and second and third generation cephalosporins constitute the highest risk of CDI in comparison to rifampicin, aminoglycosides, vancomycin, and metronidazole. According to the European Centre for Disease Prevention and Control, the total antibacterial administration for systemic use between 2019–2023 in Poland has been some of the highest in Europe [22]. These high rates of consumption continue despite the antimicrobial stewardship being practiced in healthcare institutions under government policy. The main purpose is to restrain the increasing drug resistance by developing pharmacotherapy standards for infections and using relevant prophylaxis [23]. Another reason is that the medical staff do not adhere to sanitizing and antiseptic policies. Thus, the bacteria can spread not only by their hands but also by improperly sterilized medical equipment, as *C. difficile* spores are highly resistant to chemical disinfectants and heat, allowing them to persist on surfaces and medical devices for extended periods [24]. Patients with reduced protection against pathogens, such as those with impaired immunity, cancer, diabetes, hepatocirrhosis, inflammatory bowel disease (IBD), chronic kidney disease, and the elderly (≥65 years), constitute more vulnerable population groups. Obesity, which is associated with a lower diversity of gut microbiota, and proton pump inhibitors (PPIs), which via decreasing gastric acidity change gut microbiome, create conditions favoring *C. difficile* colonization [13,24,25]. The main risk factors for primary and recurrent CDIs are presented in Figure 1.

Antibiotic therapy remains the cornerstone of treatment for CDI, despite being the primary risk factor for its development. Current guidelines from IDSA/SHEA, ESCMID, and others recommend fidaxomicin as the first-line therapy for both initial episodes and recurrences due to its superior efficacy in preventing recurrences compared to vancomycin. Vancomycin is considered an effective second-line option, particularly when fidaxomicin is unavailable or cost-prohibitive. Metronidazole, previously a common treatment, is now reserved as a third-line option for mild cases or situations where other treatments are unsuitable [26,27,28]. Recent observational studies from 2024 highlight fidaxomicin’s ability to reduce recurrence rates, although it may be less effective than metronidazole in specific recurrent cases [28]. These findings reaffirm vancomycin as a reliable alternative, with minimal observed resistance [27]. However, other studies have shown that almost 60% of *C. difficile* strains in Europe are resistant to multiple antimicrobials [29]. A study from China has found 77.85% multi-drug-resistant strains [30]. There are several possible molecular resistance mechanisms to first-line antibiotics. They may affect the cell’s wall, iron metabolism, DNA repair, or surface factors [31]. Different studies have recognized the genes of antibiotic resistance in *C. difficile*. Until 2017, 53 studies reported vancomycin resistance and 55 studies reported metronidazole resistance; however, insusceptibility to fidaxomicin was not found [32]. On the other hand, in Australia, ten diagnostic laboratories found only 5.7% vancomycin-resistant strains between 2015 and 2018 [33]. Two independent studies from Poland have tested strains collected between 1998–2003 and 2012–2014. All samples were susceptible to vancomycin and metronidazole [34,35]. It should be noted that biofilms may also play a role in the development of resistance [31]. One of the studies found that 71% of patients colonized with *C. difficile* were not permanently clear after vancomycin treatment [36].

The aim of our study was to investigate the prevalence of hypervirulent *C. difficile* RT027 and to evaluate the molecular epidemiology of the vancomycin resistance and the metronidazole resistance in the CDI patients’ stool samples collected in the years 2017–2019 in Central Teaching Hospital of Medical University of Lodz, Central Poland.

## 2. Results

**Figure 2:**
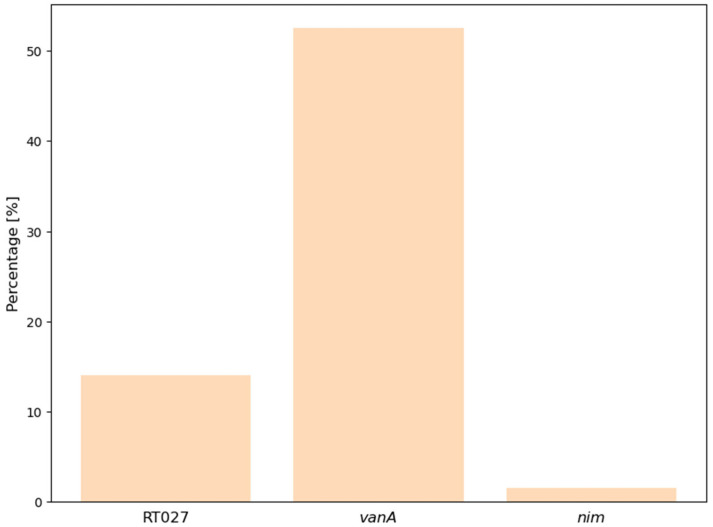
Prevalence of ribotype (RT027), *vanA*, and *nim* genes in tested stool samples (n = 200).

Out of the 200 stool samples analyzed, RT027 was detected in 14.0% of cases, *vanA* in 52.5%, and *nim* in 1.5% (Figure 2). The co-occurrence of RT027 with resistance genes, namely *vanA* and *nim*, is presented in Table 2. The *vanA* gene was observed in 50.0% of RT027-positive samples compared to 52.9% of RT027-negative samples; there was no significant difference (*p* = 0.935, Fisher’s exact test). Similarly, no statistically significant association between RT027 and the *nim* gene was found, possibly due to its low overall prevalence (*p* = 1.00).

The significant proportion of samples harboring the *vanA* gene suggests a widespread presence of vancomycin resistance in this cohort. Logistic regression analysis confirmed no significant association between RT027 and *vanA* positivity (adjusted odds ratio [OR] = 0.89, 95% confidence interval [CI]: 0.40–1.98, *p* = 0.775). Logistic regression analysis was not performed for the association between RT027 and the *nim* gene due to the low prevalence of *nim* (1.5%, n = 3) in the study population.

The study’s raw results are available in Supplementary Materials.

## 3. Discussion

CDI has been a serious public health threat worldwide for more than two decades. However, in Poland, *C. difficile* RT027 was not reported until 2008 [37,38]. In the period 2008–2010, when outbreaks of antibiotic-associated diarrhea occurred in three different hospitals in Poland, the incidence of RT027 out of 30 *C. difficile* isolates was 23.33% [38]. In one of the later Polish studies, the RT027 prevalence increased to 77.2% [39]. In our study we have not found such a high percentage of RT027; however, this may be related to the region, as in southern Poland, the CDI incidence per 100.000 people is higher than the average in Poland, which may result in a high prevalence of *C. difficile* RT027 [39]. However, our results were still higher than those obtained in the Pan-European study of *C. difficile* RT prevalence and antimicrobial resistance (AMR): 14% vs. 11.4%. The study, named “*Clostridium difficile* European Resistance” (ClosER), was performed during the years 2011–2016 and analyzed samples from 28 European countries [40].

In our study, we focused on *C. difficile* resistance to vancomycin and metronidazole, based on the presence of *vanA* and *nim* genes in biological samples received from CDI patients. In the last decades, metronidazole and vancomycin have been used as the first-line therapies in cases of CDIs. The genes encoding resistance to metronidazole (*nim*) and vancomycin (*vanA*) are carried on plasmid or chromosome and transposon 1546 (Tn1546), respectively [41].

The best-documented mechanism of vancomycin resistance is an alteration of the drug target site, i.e., modification in peptidoglycan precursors, which is mediated by the van genes. These genes, well-known in *Enterococcus* spp., have been also identified in *C. difficile* (*vanA*, *vanB*, *vanG*, *vanW*, and *vanZ* orthologs) and are associated with elevated vancomycin MICs [42].

Generally, we observed a high prevalence of the *vanA* gene, which suggests that vancomycin should not be used as a first-choice drug in CDI treatment, as is still recommended in the Polish guidelines [26]. All the more so, as CDI treatment outcomes are not usually explained in the context of AMR, because anaerobic susceptibility testing of patient isolates is not routinely performed as part of CDI diagnostics. The increasing number of reports on *C. difficile* resistance to traditional and new antibiotics justify the reevaluation of this view [31]. Although we found no association between RT027 and non-susceptibility to vancomycin, it could be related to a small study group. As reported by Eubank et al. [43], RT027 was the sole independent risk factor for reduced vancomycin susceptibility. Also, a study from Israel found relatively high non-susceptibility rates for vancomycin, which were correlated with RT027 [44].

Metronidazole, a nitro-group-containing drug, is activated within cells, generating free radicals that damage cellular components, including DNA and metalloclusters of proteins, and deplete cellular low-molecular-weight thiols [31]. The proposed mechanism of metronidazole resistance is based on the impairment of oxidoreductive metabolic pathways, and *nim* genes that encode nitroimidazole reductases, are responsible for drug inactivation.

We found *nim* genes in 1.5% of the cases studied. However, as reported by Peláez et al. [45], the lack of *nim* genes does not mean the *C. difficile* isolates are not metronidazole resistant. Other alternative metronidazole resistance mechanisms have been suggested, such as reduced uptake of metronidazole, reduced nitroreductase activity, or decreased pyruvate–ferredoxin oxidoreductase activity. However, *nim* gene-mediated resistance must be under surveillance mostly because of the location of *nim* genes on mobile genetic elements and high selective pressure coming from the indication of metronidazole administration as a first-line drug in *C. difficile* infections in certain subsets of cases (e.g., children). Polish guidelines suggest using metronidazole as a third-choice drug in the first episode of CDI [46]. The point that metronidazole is no longer recommended as first-line therapy for adults was underscored in several publications [47,48,49].

The *vanA* and *nim* genes, conferring resistance to vancomycin and metronidazole respectively, have significant ecological implications within the gut microbiome. Their presence not only complicates treatment strategies for CDI but also poses a broader threat through potential horizontal gene transfer to other potentially pathogenic bacteria. Studies have demonstrated that gene transfer events in the gut can lead to the spread of resistance determinants, thereby enhancing the resilience of pathogenic bacteria against antibiotic therapies [50,51]. Non-toxigenic *C. difficile* strains, which lack the toxin genes responsible for disease symptoms, can act as reservoirs for resistance genes like *vanA* and *nim*. These strains, prevalent in various environments including soil and water, possess mobile genetic elements capable of transferring resistance genes to toxigenic counterparts or other pathogens within the gut. This exchange exacerbates the challenge of controlling infections, as it contributes to the emergence of multidrug-resistant organisms [52,53].

During the 5-year study period of the Pan-European longitudinal study on AMR of *C. difficile*, an increase in susceptibility to metronidazole and vancomycin was observed. This could be attributed to a reduction in metronidazole and vancomycin use over the course of the study [40]. On the other hand, the increasing multi-drug resistance of *C. difficile* has been reported. In Poland, based on analysis of *C. difficile* isolates from the feces of patients from 13 hospitals, resistance to multiple antimicrobials was confirmed in 23.3% strains of RT027 (94%) [39].

The results of fidaxomicin use in reducing CDI recurrence and reducing the adverse effects of infection are promising. As found by Freeman et al. [40], no evidence of reduced susceptibility to fidaxomicin was observed following its introduction in 2011. Nevertheless, we should also take into consideration that fidaxomicin-resistant isolates have also been detected and can potentially expand [31]. Unfortunately, fidaxomicin is not being commonly used in Poland concerning economical and organizing obstacles [46]. Primary prevention is equally important. Health professionals should follow strict aseptic precautions to reduce the risk of developing resistance by the bacteria. It is also essential to adhere to the CDI control guidelines and to not cohort the infected patients but isolate them.

Because there are few antibiotic treatment options available for CDI, increasing selection pressure is being exerted towards the development of antibiotic resistance in *C. difficile*. This may lead to significant clinical implications, including the possibility of treatment failure in cases of infection. This is of particular importance in our region of Europe, as the latest report shows that increased AMR has been detected in Eastern Europe and it has been mainly associated with RT027 [19,20]. Another potential danger associated with *C. difficile* is the fact that these strains can act as a reservoir of antibiotic resistance genes and facilitate their transfer to other pathogens [54].

### Strengths and Limitations

This study provides valuable insights into the molecular epidemiology of *C. difficile* infections, particularly the prevalence of hypervirulent RT027 and co-occurrence of *vanA* and *nim* genes. One major strength of the study is the direct analysis of genetic material from whole stool samples rather than isolated *C. difficile* cultures. This approach captures the entire microbial ecosystem, reflecting real-world conditions and allowing the detection of resistance genes that could potentially be transferred horizontally and incorporated into *C. difficile* genome. This is especially relevant for genes like *vanA* and *nim*, which are known to be associated with resistance and may enhance the adaptive potential of *C. difficile*.

However, this methodological choice also poses certain limitations. The presence of resistance genes in the whole stool sample does not definitively confirm their integration into *C. difficile* genome, as they may originate from other gut microbes. Furthermore, while the study focused on the most commonly described resistance genes (*vanA* and *nim*), it is possible that other mechanisms of resistance, such as biofilm formation or mutations affecting drug targets, have not been captured. This highlights the need for future studies employing broader genomic and functional analyses to identify additional resistance mechanisms.

Despite these limitations, the study underscores the importance of monitoring genetic elements within the broad population of gut microbiota, as they represent a reservoir for potential horizontal gene transfer processing and subsequent incorporation into pathogenic strains of *C. difficile*.

## 4. Materials and Methods

A total of 200 stool samples from CDI patients, hospitalized at the Medical University of Lodz Central Teaching Hospital between the years 2017–2019, were analyzed for the presence of *C. difficile* RT027, as well as vancomycin resistance (*vanA*) and metronidazole resistance (*nim)* genes. All stool samples were previously tested (according to hospital procedure) and resulted positive for the presence of glutamate dehydrogenase (GDH) antigen and *C. difficile* A/B toxins, using TechLab C diff Quick Check Complete (TechLab, Blacksburg, VA, USA), and then were anonymized and frozen in −80 °C.

All stool samples were collected and processed in strict adherence to our hospital’s established protocols to ensure the integrity of the specimens. Immediately upon collection, each stool sample was placed in a clean, watertight container and promptly transported to the laboratory. Upon arrival, specimens were either tested immediately or stored at 2 °C to 8 °C until testing could be performed. To prevent false-negative results, samples not tested promptly were refrigerated until testing was possible, for a maximum of 18 h. This practice aligns with guidelines from the Centers for Disease Control and Prevention (CDC), which emphasize that *C. difficile* toxin degrades at room temperature and may become undetectable within two hours of stool specimen collection [55]. By adhering to these stringent handling and storage procedures, our hospital laboratory ensured the preservation of *C. difficile* toxins and antigens, thereby minimizing the risk of false-negative results during the initial antigen testing for *C. difficile* toxins A and B, as well as GDH.

Whole genetic material was isolated directly from defrosted stool samples, using QIAamp Fast DNA Stool Mini Kit (Qiagen, Hilden, Germany). Polymerase chain reaction (PCR) was performed to detect genes encoding RT027 (based on the polymorphism in the 16S-23S intergenic spacer region), vancomycin resistance (*vanA*), and metronidazole resistance (*nim*), using specific primers (see Table 3). PCR products were analyzed by gel electrophoresis with the RT027 reference strain PCR product and 100 bp marker. Gels were photographed under an ultraviolet light transilluminator BioDoc-It (Analytik Jena, Jena, Germany).

Statistical analysis was performed using Python (version 3.10) with libraries such as Pandas (version 2.2.3) for data management, SciPy (version 1.11.3) and Statsmodels (version 0.14.3) for statistical testing, and Matplotlib (version 3.8.1) for visualizations. The prevalence of ribotype and resistance genes (RT027, *vanA*, and *nim*) was calculated as percentages. The association between ribotype and the presence of the resistance gene was assessed using chi-square or Fisher’s exact tests for categorical variables. Fisher’s exact test was applied when expected counts in any cell of the contingency table were ≤ 5; otherwise, chi-square test was used. Univariate logistic regression analysis was performed to identify predictors of *vanA* and *nim* gene presence. The dependent (outcome) variable in logistic regression was the presence of the gene. Independent (predictor) variables included RT027 status. Statistical significance was set at a *p*-value < 0.05.

This study was conducted following the Good Clinical and Laboratory Practice Guidelines and the Helsinki Declaration. There was no need to obtain the consent of the Bioethics Committee to conduct the study because, in the light of the law in force in Poland, the study is not a “medical experiment”, as no patient information was included.

## 5. Conclusions

The relatively high prevalence of *vanA* in our cohort suggests a concerning level of vancomycin resistance, challenging the current treatment guidelines in Poland that still recommend vancomycin as a first-line option. Furthermore, while the *nim* gene was less prevalent, its detection emphasizes the need to monitor metronidazole resistance, particularly given the reliance on this drug in certain clinical settings. The relatively low overall occurrence of RT027 in this study contrasts with its previously reported high prevalence in other regions of Poland and Europe. This may reflect regional variations in antimicrobial use, infection control practices, or surveillance intensity.

The findings of this study highlight the prevalence of hypervirulent *C. difficile* RT027, which underscores the necessity of continuous molecular surveillance of CDI cases to guide therapeutic decisions and infection control measures.

## Figures and Tables

**Table 1:** Examples of critical *Clostridioides difficile* ribotypes [10,11,12].

Category	Ribotype	Key Characteristics
Epidemic (hypervirulent)	RT027	- produces 16 times more toxin A and 23 times more toxin B than other strains - produces a binary toxin - highly resistant to fluoroquinolones - common in hospital-acquired CDIs
	RT176	- closely related to RT027 (bears the same mutation) - produces a binary toxin - first cases of RT176-associated CDI were described in 2008 in Poland - regional specificity: Czech Republic - rapid nosocomial spread through Europe
	RT078	- produces toxins A and B, and a binary toxin - resistant to fluoroquinolones and erythromycin - common in community-acquired CDIs in a younger population - found in animals (suggesting a foodborne interspecies transmission)
	RT126	- closely related to RT078 (bears the same mutation) - produces toxins A and B, and a binary toxin - potential zoonotic spread - frequently resistant erythromycin, moxifloxacin, and tetracycline
	RT181	- emerging ribotype with high antimicrobial resistance to moxifloxacin, clindamycin, and rifampin - shares genetic similarities with RT027 - increasingly reported in Eastern Europe
Non-epidemic (endemic in almost all European countries)	RT001/072	- frequently isolated from environmental sources, such as freshwater sediments - not linked to severe infections
	RT014/020	- common in clinical settings - not typically associated with widespread outbreaks

**Table 2:** Co-occurrence of RT027, *vanA*, and *nim* genes in tested stool samples (n = 200).

	*vanA*	Absent	Absent	Present	Present	TOTAL
	*nim*	Absent	Present	Absent	Present	
RT027	Absent	79	2	90	1	172
	Present	14	0	14	0	28
	TOTAL	93	2	104	1	200

**Table 3:** Details on oligonucleotide primers used in the study.

Gene	Primer Sequences	References
16S-23S	Forward: 5′-GTGCGGCTGGATCACCTCCT-3′ Reverse: 5′-CCCTGCACCCTTAATAACTTGACC-3′	Bidet et al. [56]
*vanA*	Forward: 5′-GGGAAAACGACAATTGC-3′ Reverse: 5′-GTACAATGCGGCCGTTA-3′	Phukan et al. [57]
*nim*	Forward: 5′-ATGTTCAGAGAAATGCGGCGTAAGCG-3′ Reverse: 5′-GCTTCCTTGCCTGTCATGTGCTC-3′	Trinh et al. [58]

## Data Availability

The original contributions presented in this study are included in the Supplementary Material. Further inquiries can be directed to the corresponding author.

## Supplementary Materials

The following supporting information can be downloaded at: https://www.mdpi.com/article/10.3390/antibiotics14030219/s1. Supplementary File S1. Excel sheet with raw data.
